# Heart Uptake of [^18^F]Fluoro-4-Thia-Oleate in a Non-Alcoholic Fatty Liver Disease Mouse Model

**DOI:** 10.3390/ph15121577

**Published:** 2022-12-17

**Authors:** Junfeng Li, Weidong Hu, Jiangling Peng, Patty Wong, Fouad Kandeel, Tove Olafsen, John E. Shively

**Affiliations:** 1 Arthur Riggs Institute of Diabetes, Endocrinology and Metabolism, Beckman Research Institute of the City of Hope, Duarte, CA 91010, USA; 2 Department of Radiation Oncology, City of Hope National Medical Center, Duarte, CA 91010, USA; 3 Small Animal Imaging Core, Shared Resources, City of Hope, Duarte, CA 91010, USA

**Keywords:** non-alcoholic fatty liver disease, CEACAM1, [^18^]F-fluoro-4-thia-oleate, PET/MRI

## Abstract

The world-wide high incidence of non-alcoholic fatty liver disease (NAFLD) is of concern for its progression to insulin resistance, steatohepatitis and cardiovascular disease (CVD). The increased uptake of fatty acids in critical organs plays a major role in NAFLD progression. Male *Ceacam1^−/−^* mice that develop NAFLD, insulin resistance and CVD on normal chow are a potential model for studying the dysregulation of fatty acid uptake. [^18^F]fluoro-4-thia-oleate ([^18^F]FTO) was chosen as a fatty acid reporter because of its higher uptake and retention in the heart in an animal model of CVD. Male wild-type (WT) or *Ceacam1^−/−^* mice fasted 4–6 h were administered [^18^F]FTO i.v., and dynamic PET scans were conducted in an MR/PET small animal imaging system along with terminal tissue biodistributions. Quantitative heart image analysis revealed significantly higher uptake at 35 min in *Ceacam1^−/^^−^* (6.0 ± 1.0% ID/cc) vs. WT (3.9 ± 0.6% ID/cc) mice (*p* = 0.006). Ex vivo heart uptake/retention (% ID/organ) was 2.82 ± 0.45 for *Ceacam1^−/^^−^* mice vs. 1.66 ± 0.45 for WT mice (*p* < 0.01). Higher kidney and pancreas uptake/retention in *Ceacam1^−/^^−^* was also evident, and the excretion of [^18^F]FTO into the duodenum was observed for both WT and *Ceacam1^−/^^−^* mice starting at 10 min. This study suggests that the administration of [^18^F]FTO as a marker of fatty acid uptake and retention may be an important tool in analyzing the effect of NAFLD on lipid dysregulation in the heart.

## 1. Introduction

Non-alcoholic fatty liver disease (NAFLD) in western countries affects about 25% of adults and represents a major health threat since its progression can affect other critical organs such as the heart and kidneys [1,2]. The association of NAFLD with obesity, insulin resistance, metabolic disease and type 2 diabetes (T2D) further increases the risk of cardiovascular disease [3,4,5]. Although the underlying mechanisms leading to NAFLD are debated, it is generally agreed that a higher-than-normal uptake of free fatty acids in organs over time contributes to the disease [6]. Thus, many patients with NAFLD may develop cardiovascular disease (CVD) at a late stage and treatment modalities are lacking [7]. Increased fatty acid uptake along with decreased fatty acid metabolism may lead to cardiomyopathies, including enlarged heart, decreased cardiac performance and eventual heart failure [8]. CVD involving the coronary arteries is also an increased risk of death due to general lipid dyslipidemia and atherosclerosis associated with NAFLD [9]. Chronic fatty liver can lead to non-alcoholic steatohepatitis (NASH) and the subsequent development of liver cancer [10]. Another hallmark of NAFLD is increased visceral adiposity, suggesting a global dysregulation of fat storage [11]. There is also a preponderance of the disease in males, while in females the disease is seen mostly in post-menopausal women [12]. The goal of this study was to determine if a PET probe for fatty acid cardiac uptake would differentiate normal hearts from the hearts of animals with early NAFLD.

Researchers have relied on genetic or environmental animal models to study NAFLD [13]. The genetic models using obese ob/ob or db/db mice exhibit many features of the human disease but lack others, including the development of CVD. A similar situation exists for models requiring the extensive use of high fat or high sucrose diets for 16–20 weeks [14]. In this study, we chose the *Ceacam1^−/−^* model that develops NAFLD, insulin resistance, obesity and CVD in male *Ceacam1^−/−^* mice on normal chow starting at the age of 12–15 weeks and progressing to overt symptoms by ages >20 weeks [15,16,17,18,19]. Since CEACAM1 plays an inhibitory role in the immune system [20], its loss or the signaling defects associated with CEACAM1 in the liver may partially explain the inflammatory nature of NAFLD. In this respect, we have recently shown that human CEACAM1 directly associates with the fatty acid uptake receptor CD36 in human HepG2 hepatocytes [21] and is phosphorylated by a combination of protein kinase A (PKA) and glycogen synthase kinase 3b (GSK3b) [22]—key kinases that regulate glucagon [23] and insulin signaling [24,25]. In addition, the deletion of CEACAM1 in mice leads to an elevation of CD36 and pAMPK expression [26]—proteins that are co-regulated in skeletal and heart muscle [27]. In terms of CVD, endocardiography revealed increased septal wall thickness in male *Ceacam1^−/−^* mice but not in CEACAM1 liver-reconstituted male *Ceacam1^−/−^* mice [19]. Inflammation, leukocyte recruitment and fibrosis in the aorta of male *Ceacam1^−/−^* mice on normal chow are further indications of CVD [28]. Taken together, these studies indicate that male *Ceacam1^−/−^* mice are a good model for the development of CVD in fatty liver disease, in which fatty acid uptake and metabolic dysfunction are key factors. As a starting point in probing the role of fatty acid uptake in NAFLD, we compared male wild-type vs. male *Ceacam1^−/−^* mice on normal chow for fatty acid uptake using a ^18^F-labeled fatty acid probe.

Altered fatty acid uptake and management in critical organs is a likely starting point for both the diagnosis and mechanistic insights into the origins and progress of NAFLD. Similar to studies with altered glucose uptake and management in diseases such as diabetes and cancer, the development of a suitable non-metabolizable tracer is essential for imaging. An example is ^18^F-fluorodexoyglucose ([^18^F]FDG) that is taken up by the same transporters as glucose but does not enter glycolysis. [^18^F]FDG has become the standard in positron emission tomography (PET) imaging for identifying tissues such as visceral fat that have altered glucose uptake [29]. Since the half-life of ^18^F is 109.7 min, [^18^F]FDG can be prepared off site, locally administered, and PET imaging can be performed in a reasonable clinical workflow. However, in the case of fatty acid imaging, early fatty acid PET tracers such as [^11^C]palmitate were radiolabeled with ^11^C, a radionuclide with a half-life of only 20.3 min [30]. This probe limits its clinical application due to the rapid metabolism of palmitate and the short half-life of ^11^C that requires an on-site cyclotron for production and rapid chemical incorporation into fatty acid precursors [31]. The development of ^18^F-labeled fatty acids tracers resulted in the first generation 14-[^18^F]fluoro-6-thia-heptadecanoic acid (FTHA) that satisfied some, but not all, of the requirements for an ideal tracer [32]. The eventual selection of an oleic-acid-based tracer [^18^F]FTO over the palmitic acid derivative [^18^F]FTP was especially useful in cardiac imaging [33]. The reasoning behind this selection was the increased selectivity for oleic acid over palmitic acid uptake in the heart [34] and the observed higher retention of [^18^F]FTO vs. [^18^F]FTP in a rat model of cardiomyopathy [33]. Here, we reported the use of [^18^F]FTO as a tracer to evaluate cardiac imaging in the *Ceacam1^−/−^* animal model that develops fatty liver disease and CVD.

## 2. Results

### 2.1. Initial [^18^F]FTO Uptake Studies in WT Male Mice

On the basis of the study by Degrado et al. [33], the oleic acid derivative [^18^F]FTO was chosen as an appropriate fatty acid uptake probe that is only slowly metabolized compared to the palmitic acid derivative [^18^F]FTP [35]. The initial [^18^F]FTO uptake studies were performed in 44-week-old WT male mice to determine the appropriate organ uptake kinetics and if absolute uptake amounts were sufficient for further studies. Older male mice were chosen because of the known tendency of the C57/B6 strain of mice to develop fatty liver disease with age [36]. The mice were morning-fasted prior to the injection of the probe as an appropriate fasting strategy for nocturnal animals. Supplementary Figure S1 shows two-hour dynamic PET scans of a mouse following administration of 3.54 MBq [^18^F]FTO. The sagittal and coronal maximum intensity projection (MIP) images are shown in the top panel. Initial uptake was seen from the site of tail vein injection into the vena cava and heart (0.25 min) followed by rapid distribution into the liver (0.5 min). By 30 min, symmetrical distribution into large bone joints, spine and jaw was observed, along with excretion into the kidneys and duodenum. Continued imaging out to 120 min revealed whole body clearance with retention into large bony joints, the jaw and spine. Measurable spinal uptake started at approximately 20 min and continued out to 120 min post-injection (p.i). Jaw uptake as well as uptake into the snout was similar to spine uptake. The hot spots seen in the extremities at 120 min p.i. were shoulder and knee joints. MR imaging was performed at the same time as PET imaging to allow anatomical registration of the PET images. The bottom panel in Supplementary Figure S1 shows the sagittal and coronal multiplanar reconstructed (MPR) PET/MR overlay images of the same animal.

A time–activity curve (TAC) was generated from the dynamic scan of mouse 1 by drawing ROIs on the images. The plot in Supplementary Figure S2 reveals rapid clearance from the blood (<1 min p.i.) with heart retention showing a plateau from 2–50 min p.i. and a slow clearance thereafter. The kinetics of uptake/retention in the heart was nearly coincident with whole heart imaging. Liver uptake was very rapid (0.5–10 min p.i.) with an exponential clearance out to 120 min p.i. The kidneys followed a similar kinetics compared to the liver at about 50% of the maximum liver levels. Linear leg joint uptake began at about 5 min p.i. and plateaued at 50–60 min p.i., while linear spine uptake was observed from 10–50 min p.i. followed by a slow rise to plateau at about half the level of the leg joint uptake. Muscle and bladder uptakes were negligible. With the exception of bone, all tissues were below 5% ID/cc at 2 h. The half-lives of the tracer in the blood were calculated from the time–activity curve to be 0.2256 min (fast) and 166.2 min (slow) for the initial distribution and elimination phase, respectively. Since only minor changes were seen in the period 30–120 min p.i., a shortened dynamic imaging time span was chosen for subsequent studies to allow imaging of additional mice for a given preparation of [^18^F]FTO. No further studies on 44-week-old WT mice were performed since our goal was to study the early stages of NAFLD in larger numbers of younger *Ceacam1^−/−^* mice.

### 2.2. [^18^F]FTO Uptake Studies in WT vs. Ceacam1^−/−^ Male Mice

**Figure 1:**
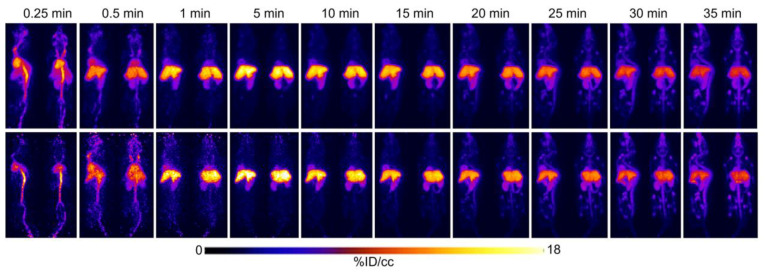
Comparative PET images of 15–18-week-old, fasted WT vs. *Ceacam1^−/−^* mice administered [^18^F]FTO followed by a 35 min dynamic scan. Top panel: male WT mouse injected with 6.7 MBq of [^18^F]FTO. Bottom panel: male *Ceacam1^−/−^* mouse injected with 2.7 MBq of [^18^F]FTO. Selected time points are shown from a representative animal (*n* = 4 per group).

**Figure 2:**
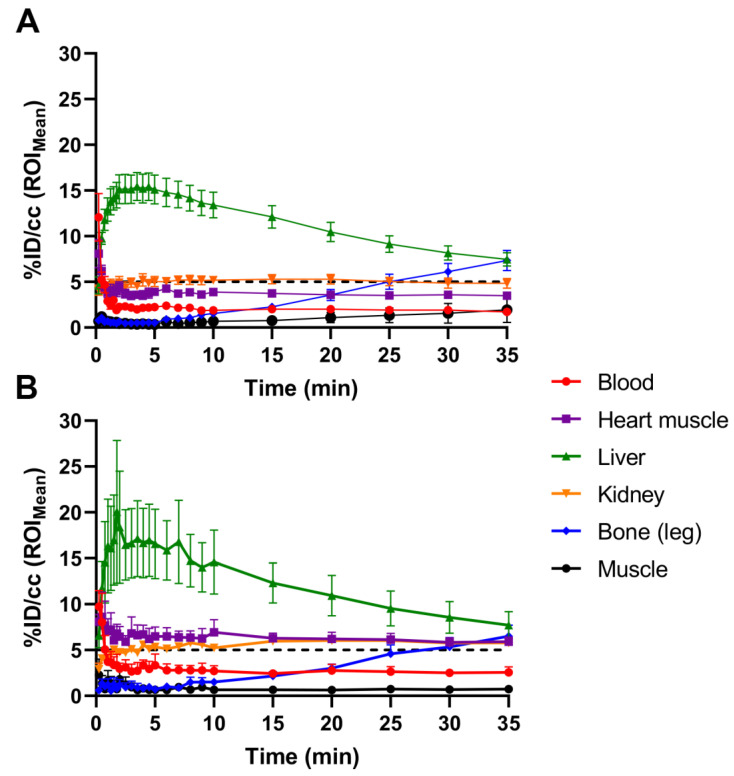
Comparative time–activity curves of [^18^F]FTO uptake in WT vs. *Ceacam1^−/−^* male mice. The % ID/cc ± SEM (*n* = 4) are shown for WT (**A**) and *Ceacam1^−/−^* mice (**B**).

[^18^F]FTO uptake in WT vs. *Ceacam1^−/−^* male mice was compared by serial imaging and terminal ex vivo biodistribution analyses. The body weights of the WT and *Ceacam1^−/−^* mice (*n* = 5) were similar for 18-week-old WT and 15-week-old *Ceacam1^−/−^* mice used in the study. Figure 1 shows representative PET images of a WT vs. a *Ceacam1^−/−^* mouse over a 35 min time span at selected time points. Although the images are similar for the WT and *Ceacam1^−/−^* mice, time–activity curves (Figure 2) show heart uptake (*n* = 4) in the *Ceacam1^−/−^* mice (7.0 ± 0.4% ID/cc) is higher vs. WT mice (4.0 ± 0.2% ID/cc) at 35 min. Liver activity reached a maximum uptake at approximately 5 min p.i., ~18% ID/cc in the WT mouse and ~28% ID/cc in the *Ceacam1^−/−^* mouse, which subsequently declined to 9.4 and 11.9% ID/cc, respectively, at 35 min. In addition, the activity in the kidney was higher in the *Ceacam1^−/−^* mouse (6.3 vs. 5.3% ID/cc).

**Figure 3:**
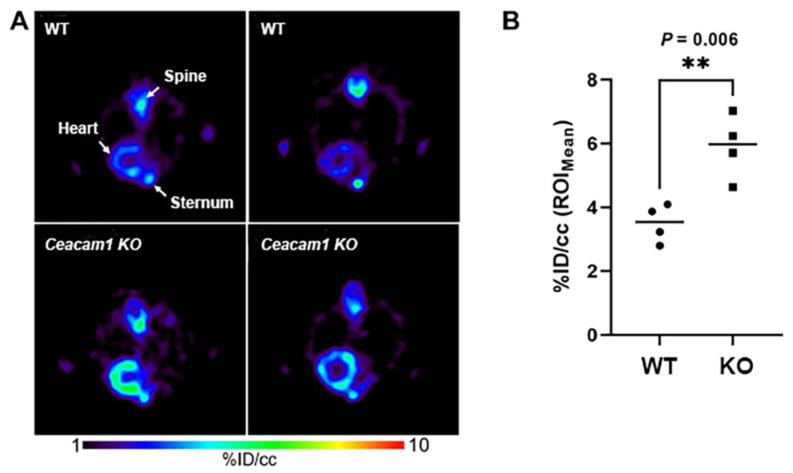
Differential heart muscle uptake/retention of [^18^F]FTO in WT and *Ceacam1^−/−^* mice. (**A**) Representative transverse MPR PET images at 35 min p.i. of WT and *Ceacam1^−/−^* mice hearts at age 15–18 weeks. (**B**). ROI quantification of heart muscle in the two groups (*n* = 4 per group, ** *p* = 0.006).

The mean activity concentration in the tissues of WT and *Ceacam1^−/−^* mice shown in Figure 2. *Ceacam1^−/−^* mice also reveal higher activity in kidney that remains higher over the entire time course. In addition, initial liver uptake/retention appears to be slightly higher in *Ceacam1^−/−^* mice, but by 35 min there is no significant difference between the two groups due to the high variability in the *Ceacam1^−/−^* group. Transverse PET images of the mice in the heart confirmed the higher activity concentration in the heart muscle of *Ceacam1^−/−^* mice (Figure 3A). Quantitative heart image analysis (*n* = 4) at 35 min of *Ceacam1^−/−^* (6.0 ± 1.0% ID/cc) vs. WT (3.9 ± 0.6% ID/cc) WT mice (Figure 3B) gave a significantly higher uptake/retention ratio of 1.54 (*p* = 0.006).

Tissue biodistributions performed at 40 min p.i. confirmed the differential tissue uptakes observed by quantitative PET imaging. The radioactive uptake in the heart (Table 1) was significantly higher in the *Ceacam1^−/−^* mice (2.82 ± 0.45% ID) vs. WT (1.66 ± 0.45% ID) resulting in an average organ ratio of 1.70 which was similar to the % ID/cc ratio calculated by quantitative PET image analysis (Figure 3). In addition, the comparative heart biodistribution calculated for % ID/g (Supplementary Figure S3) was similar to the quantitative image analysis reported in % ID/cc (Figure 3). Although liver biodistribution analysis appeared higher in *Ceacam1^−/−^* mice, it was not statistically significant, again likely due to high individual variability among the *Ceacam1^−/−^* mice (Table 1), in agreement with the TAC analysis (Figure 2). Combining liver plus gall bladder (liver/gallbladder) values were also not statistically significant. Higher uptake/retention in the kidneys and pancreas was apparent in *Ceacam1^−/−^* mice with an average ratio of 1.5 for both organs. In addition, excretion into the small intestine met the criteria for significance (*p* < 0.05) between the two groups.

## 3. Discussion

There is a growing consensus that fatty liver disease increases the risk of cardiomyopathy and that this risk is associated with increased uptake of fatty acids into the heart [37]. Since fatty acids play a large role in heart disease associated with insulin resistance and metabolic disease [7], development of appropriate fatty acid tracers in animal models is an essential first step in moving the tracers to the clinic. We chose the male *Ceacam1^−/−^* model that develops fatty liver disease and CVD in an age related manner on normal chow, since fatty liver disease is known to occur in both obese and nonobese adults [38], is age related, and has a sex prevalence in males [39]. In addition, the *Ceacam1^−/−^* mouse model is appropriate since these mice exhibit insulin resistance, metabolic disease and cardiomyopathy [19]. Nonetheless, this model, like other genetic models of NAFLD, can be criticized for a lack of evidence for genetic mutations in the human CEACAM1 gene that correlate with NAFLD, T2D, or CVD. However, given the high expression of CEACAM1 in the liver and its co-expression with fatty acid translocase CD36 [21] and insulin receptor [17], it is likely that downstream signaling pathways involving CEACAM1 are involved in the disease. Thus, the *Ceacam1^−/−^* model opens a window into the effects of loss of CEACAM1 signaling in the liver.

As a tracer we chose [^18^F]FTO, an oleic acid derivative, as an uptake marker for its superior heart retention over the palmitic acid tracer [^18^F]FTP [33] and the longer half-life of ^18^F over ^11^C-fatty-acid-based imaging. Factors that may affect its uptake into organs include altered levels of serum free fatty acids due to either fasting conditions or other pathologies associated with NAFLD. In terms of fasting, similar to glucose levels, serum levels of free fatty acids in C57/B6 mice remain relatively constant, in the range of 0.5–0.8 mM [40,41]. Although there was an increase in serum free fatty acids in 6 month old male *Ceacam1^−/−^* vs. WT mice on a normal diet from 0.7 to 1.0 mM [42], lower rather than higher levels would be expected to have a greater effect on free fatty acid organ uptake. In terms of organ weights, the heart weights were similar (0.46 g) for WT and *Ceacam1^−/−^* mice [43]. Thus, it is likely that the increased heart uptake of [^18^F]FTO in *Ceacam1^−/−^* vs. WT mice is a real indicator of heart pathology. Nonetheless, the use of [^18^F]FTO as a tracer for organ fatty acid uptake is not without its drawbacks. The bone uptake indicates that metabolic release of [^18^F]fluoride ion occurred, a common issue with [^18^F]-labeled tracers. Since the blood stability of this probe is good, metabolic defluorination in tissues followed by [^18^F]fluoride ion uptake is likely responsible [33]. Thus, there is a real need for the further development of metabolically stable fatty acid tracers that can compete with the clinical use of [^18^F]FDG for glucose uptake. The further development of PET probes for fatty acid uptake in NAFLD have been recently reviewed [44].

In addition to the heart, there was a statistical difference in the pancreas between *Ceacam1^−/−^* and WT animals. Although the magnitude of the uptake was low compared to the heart, the biological significance of fatty acid uptake into the islet cells of the pancreas is important [45], suggesting further studies are warranted to determine if the uptake is primarily in beta-cells. Higher uptake into the kidneys of *Ceacam1^−/−^* mice is also important in that NAFLD accelerates chronic kidney disease [46]. There was a strong suggestion that liver uptake was higher in *Ceacam1^−/−^* mice, but the individual variation was high in this group, suggesting that variables such as fasting time, lipid content, and hormonal status need to be examined. The data also demonstrate excretion of the tracer into the duodenum. Since this result may be due to either metabolic or phospholipid excretion via bile, further studies are warranted.

## 4. Materials and Methods

### 4.1. Animal Studies

This study was carried out in strict accordance with the recommendations of the Guide for the Care and Use of Laboratory Animals of the National Institutes of Health. The protocol (number 11033) was approved by the Institutional Animal Care and Use Committee (IACUC) of the City of Hope, an AAALAC approved facility (assurance number A3001-01). *Ceacam1^−/−^* mice were generated by Dr. Nicole Beauchemin and coworkers [47]. Wild type (WT) C57/BL6 mice were purchased from Jackson laboratory (Bar Harbor, ME, USA).

#### 4.1.1. Radiosynthesis

[^18^F]FTO and [^19^F]FTO were synthesized as previously described with slight modifications [33]. The “cold” standard was confirmed by mass spectrometry ([^19^F]FTO: *m/z* 357.18 for [M + K]^+^, C_17_H_31_KFO_2_S, calculated [M + K]^+^ 357.16). Typically, for synthesis of [^18^F]FTO, 7.4 GBq (200 mCi) of ^18^F-fluoride (PETNET Solutions Inc., Culver City, CA, USA) was passed through a QMA cartridge (Waters, Milford, MA, USA). Cartridge trapped ^18^F-fluoride was eluted into the reactor tube by a solution containing Kryptofix 2.2.2 (18 mg), acetonitrile (0.9 mL), and K_2_CO_3_ (4 mg) in water (0.2 mL). Eluted ^18^F-fluoride was dried by azeotropic distillation and, a solution of the methylester bromide precursor, namely methyl (Z)-3-((14-bromotetradec-5-en-1-yl)thio)propanoate (2 mg, 5.1 µmol) in acetonitrile (0.5 mL) was added, and heated at 75 °C for 15 min. The reactor tube was cooled down, and 0.3 mL of 0.2 N KOH in acetonitrile (0.5 mL) was added and subsequent hydrolysis of the resulting ^18^F-fluoroester was performed at 90 °C for 4 min. After cooling down, the mixture was acidified with acetic acid and the product was purified on a Luna C-18 (5 µm, 10 × 250 mm) semipreparative column (Phenomenex, Torrance, CA, USA), monitored at 210 nm and radioactivity by UV-HPLC/Radio system of Synthra RNplus module. The mobile phase was acetonitrile/water/trifluoroacetic acid (90:10:0.05 *v*/*v*/*v*), and flow rate was 5 mL/min. The retention time for the desired product was ~5.5 min. The ^18^F-fluoro–fatty acid fraction was collected, diluted in 40 mL of water and trapped on a C-18 Sep-Pak cartridge (Waters). Following washing of the Sep-Pak cartridge with 10 mL of water, the product was eluted in 1.5 mL of ethanol. The volume of the ethanol solvent was removed under a nitrogen stream at 40 °C and then formulated in ~0.5–1% albumin in isotonic NaCl solution and sterile filtered through a 0.22-µm filter. The total synthesis time was ~70 min. QC was conducted on a Luna C-18 (5 µm, 4.6 × 250 mm) analytical column (Phenomenex) with the same mobile phase at 1.5 mL/min. The published protocol was followed for the synthesis of the reference ^19^F-FTO [33]. Reference [^19^F]FTO co-migration with [^18^F]FTO was confirmed by RP-HPLC (retention time was ~4.6 min). The overall radiochemical purity and yield of ^18^F-FTO were >99% and 12~15% (decay-corrected to EOS), respectively.

#### 4.1.2. PET/MR Imaging and Biodistributions

The PET and MR experiments were carried out on 7T PET/MR small animal imaging system (MR Solutions, Guildford, UK). Anesthetized mice that had been fasted for 4-6 h, were placed in a prone position on the imaging bed, and the mice isocenter position were achieved through adjusting the bed position using a 2D T1-weighted fast spin-echo scan in coronal plan. Mouse body quadrature bird cage RF coil with effective diameter of 38 mm and length of 70 mm was used for all scans. Mice were kept warm by the Minerve multistation temperature control unit (Esternay, France) throughout the experiment. The respiration rate was maintained at approximately 40/min with 2–4% isoflurane in oxygen and monitored by an MR-compatible small animal monitoring and gating system (SA Instruments, Inc., Stony Brook, NY, USA) through a respiration pad taped on the back of the mice. Single doses ranging from 2.7 to 10.2 MBq (73.5 to 275.5 µCi, 200 µL) of [^18^F]FTO in 1% serum albumin-saline was injected through a tail vein catheter. Mice were subjected to both 0–35 and 0–120 min dynamic PET scans. Three-dimensional FLASH (fast low angle shot) MR imaging with respiration gating were carried out using a TR of 30 ms, a TE of 4 ms, a flip angle of 40°, a FOV of 80 × 40 × 28 mm, and in coronal plane matrices of 256 × 128. A phase encoding of 128 steps was used in the third dimension giving section thickness of 0.22 mm. The number of excitations was 4 with a total scan time of 33 min. Acquired images were 3D-OSEM reconstructed (3 iterations, 0.28 mmm voxel size) by Preclinical Scan Software (MR Solutions) and were stored in DICOM format.

Following imaging, organs/tissues of interest (blood, heart, lung, liver, gallbladder, spleen, stomach, kidneys, pancreas, duodenum, small intestine, cecum, large intestine, brown fat, muscle, bone, and brain) were harvested from both WT and *Ceacam1^−/−^* mice (*n* = 5 per group). Organs/tissues were weighted and counted in a Wizard2 gamma counter (PerkinElmer Health Sciences Inc., Shelton, CT, USA) with three tubes containing 1% of the injected dose (standards) and three empty tubes (background). Radioactive uptakes were calculated and reported as percentage injected dose per gram (% ID/g) or % ID per organ.

#### 4.1.3. Image Analysis

PET images were displayed in both VivoQuant v3.5 post-processing software (Invicro, Needham, MA, USA) and in the Medical Imaging Data Examiner (AMIDE) software [48]. A Gauss post-reconstruction filter (FWHM = 1 mm) was applied to the PET images displayed in VivoQuant. Image analysis and quantification was conducted in AMIDE. The injected dose, decayed to scan start and time of injection, was entered into the software and ellipsoid regions of interests (ROIs) the ranged from 1.5 × 1.5 × 1.5 mm to 4 × 4 × 4 mm were drawn over tissues of interest. The calculated activity concentrations (% ID/cc) in the tissues were plotted as time–activity curves. PET images were co-registered with MR images for anatomical reference.

#### 4.1.4. Statistical Analysis

Tissue radioactive uptake values in WT and *Ceacam1^−/−^* mice were compared using two-tailed, unpaired Student’s *t* test. A *p* value of <0.05 was considered statistically significant. A non-linear fit two-phase decay curve at 95% confidence interval was used to calculate the blood clearance. The GraphPad Prism software (version 9.00 for Windows, GraphPad Software, Inc., San Diego, CA, USA) was used for all statistical analysis.

## 5. Conclusions

The fatty acid tracer [^18^F]FTO demonstrates high heart uptake in a murine model of NAFLD compared to sex and weight matched WT controls. Given the association of increased fatty acid uptake in the heart of patients with cardiomyopathy, this tracer has the potential to assess the risk of cardiomyopathy in human fatty liver disease.

## Figures and Tables

**Table 1:** Organ biodistribution (% dose/organ) of [^18^F]FTO in WT mice and *Ceacam1^−/−^* mice ^1^.

Organ/Tissue	WT Mice	*Ceacam1^−/−^* Mice	*p* Value
Heart ^2^	1.66 ± 0.45	2.82 ± 0.45	<0.01
Lung	0.32 ± 0.08	0.45 ± 0.06	0.02
Pancreas	0.25 ± 0.02	0.52 ± 0.24	0.04
Spleen	0.13 ± 0.03	0.14 ± 0.03	0.71
Kidneys	5.19 ± 0.49	8.76 ± 1.81	<0.01
Liver ^3^	15.33 ± 2.80	15.23 ± 4.17	0.96
Liver/gallbladder	15.55 ± 2.73	15.49 ± 4.24	0.98
Stomach	0.33 ± 0.08	0.45 ± 0.10	0.07
Duodenum	3.34 ± 0.52	2.96 ± 0.51	0.28
Small intestine	1.52 ± 0.03	2.17 ± 0.20	<0.01
Cecum	0.22 ± 0.04	0.24 ± 0.06	0.41
Large intestine	0.36 ± 0.07	0.45 ± 0.20	0.39
Gallbladder	0.22 ± 0.13	0.26 ± 0.23	0.71
Brown fat	0.31 ± 0.03	0.27 ± 0.08	0.33
Brain	0.42 ± 0.07	0.32 ± 0.09	0.07

^1^*n* = 5 per group, values are mean ± SD. Age of WT mice 18 weeks, Age of *Ceacam1^−/−^* mice 15 weeks. Animals were of similar weights: 31 ± 1 g. ^2^ Average weight of WT or *Ceacam1^−/−^* hearts: 0.14 ± 0.02 g. ^3^ Average weight of WT livers: 1.30 ± 0.05 g; *Ceacam1^−/−^*, 1.14 ± 0.06.

## Data Availability

Data is contained within article and Supplementary Material.

## Supplementary Materials

The following supporting information can be downloaded at: https://www.mdpi.com/article/10.3390/ph15121577/s1, Figure S1: PET and MR images of a representative male WT mouse subjected to 2 h dynamic scans with [^18^F]FTO; Figure S2: Time-activity curves of a representative male WT mouse subjected to 2 h dynamic scan with [^18^F]FTO; Figure S3: Comparative biodistributions of [ ^18^F]FTO in WT vs. *Ceacam1^−/−^* mice.
