# Supplementary material for: Heart Uptake of [18F]Fluoro-4-Thia-Oleate in a Non-Alcoholic Fatty Liver Disease Mouse Model
Source: Pharmaceuticals (Basel). 2022 Dec 17;15(12):1577. doi: 10.3390/ph15121577 (PMC9784886; doi:10.3390/ph15121577)
Supplement: Supplementary file 1 [file pharmaceuticals-15-01577-s001.zip › pharmaceuticals-2065523-supplementary.pdf]

Heart Uptake of [^{18}F]Fluoro-4-Thia-Oleate in a Non-Alcoholic Fatty Liver Disease Mouse Model

Junfeng Li, Weidong Hu, Jiangling Peng, Patty Wong, Fouad Kandeel, Tove Olafsen and John E. Shively

Supplementary data.

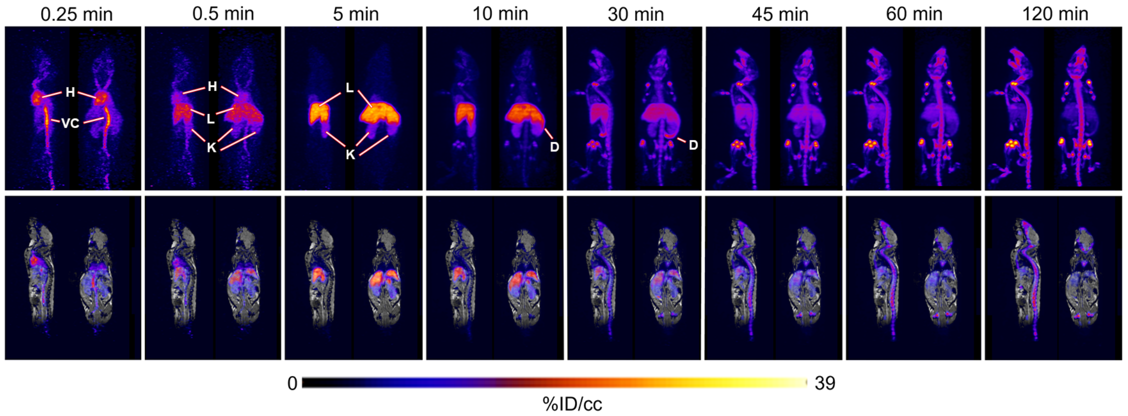

Supplementary Figure S1. PET and MR images of a representative male WT mouse subjected to 2 hr dynamic scans with [^{18}F]FTO. Mice (males, N = 2, 44 week old) were fasted for 6 hours prior to tail vein injection of 3.54 MBq of [^{18}F]FTO. **Top panel**: Sagittal and coronal MIP PET images. **Bottom panel**: Sagittal and coronal MPR PET/MR overlay images. Selected time points along with organs (H= heart, VC= vena cava, K= kidney(s), D=duodenum) are shown. Images are representative of two mice scanned.

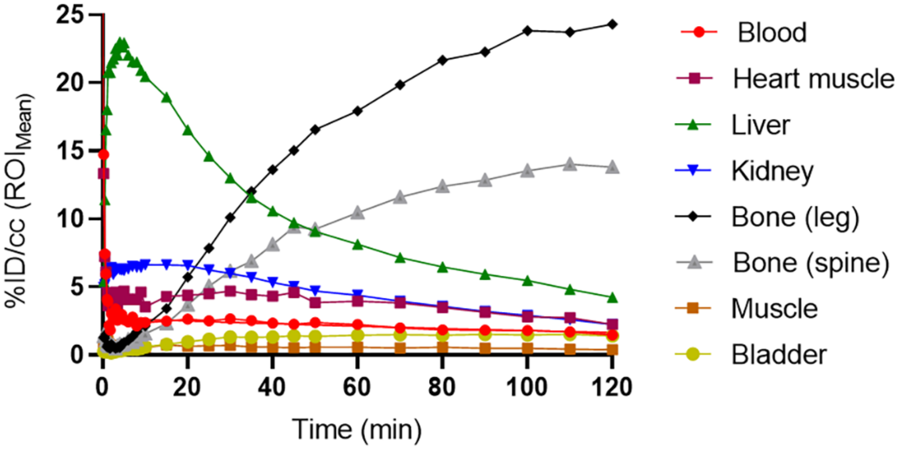

Supplementary Figure S2. Time-activity curves of a representative male WT mouse subjected to 2 hr dynamic scan with [^{18}F]FTO. The curves were generated from the 120-minute dynamic PET scan of the male WT mouse in Figure 1 injected with 3.54 MBq of [^{18}F]FTO.

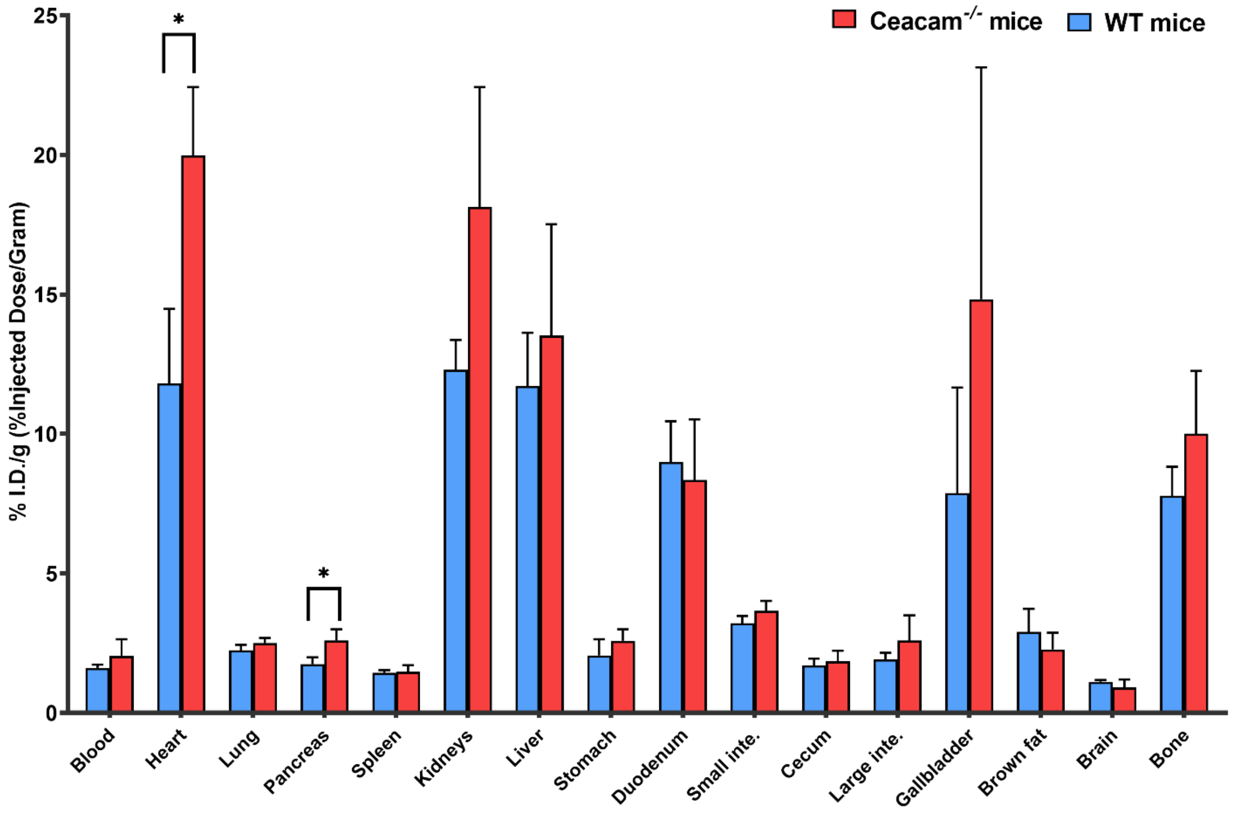

Supplementary Figure S3. **Comparative biodistributions of [^{18}F]FTO in WT vs *Ceacam$^{-/-}$* mice**. Tissue biodistributions performed at 40 minutes following injection of 2.72~6.70 MBq (73.5~189 µCi) of [^{18}F]FTO into weight matched (15-18 week old) male WT (blue) or *Ceacam$^{-/-}$* mice (red) (N = 5). * $P < 0.01$.
